# Supplementary material for: Engagement in HIV services over time among young women who sell sex in Zimbabwe
Source: PLoS One. 2022 Jun 28;17(6):e0270298. doi: 10.1371/journal.pone.0270298 (PMC9239457; doi:10.1371/journal.pone.0270298)
Supplement: Attachment — Submitted filename: Review_Zimbabwe.docx [file pone.0270298.s001.docx]

This manuscript evaluated Engagement in HIV services over time among young women who sell sex in Zimbabwe. Authors provided reasonable background information of the subject matters and justified the relevance of the study.

Comment:

Abstract

Define RDS on its first appearance

Main article

Page 7, line 122 dried blood spot (D\BS), I am not sure you wanted the back slash

Page 7, line 125, Better to say viral load suppression but not undetectable. The lower limit detection of viral load for most machines is set at 50, 40, 20. At 1000, you still detect viruses

Table1:

I will be better to rea arrange the columns such that

for 2013 arrange as 18-24 vs. 25 or older followed by its corresponding P-value. Do the same for 2016

Page 15 line 192: A couple of areas need to change verbs to past tense eg we compare, we see etc. This is true in some other areas as well

Page 15: There is title for the figure, however the figure is at the end. You may cite in the text and let the figure at the end, however, you also have tables in the text. Perhaps you should also move the figure up.

Figure: Need to improve its quality
