# Supplementary material for: Engagement in HIV services over time among young women who sell sex in Zimbabwe
Source: PLoS One. 2022 Jun 28;17(6):e0270298. doi: 10.1371/journal.pone.0270298 (PMC9239457; doi:10.1371/journal.pone.0270298)
Supplement: Attachment — Submitted filename: Response to reviewer comments, 2May22.docx [file pone.0270298.s002.docx]

**Response to reviewers' comments:**

**We thank the reviewer for their comments and suggestions. We have included a point-by-point response to their review in bold below.**

*This manuscript evaluated Engagement in HIV services over time among young women who sell sex in Zimbabwe. Authors provided reasonable background information of the subject matters and justified the relevance of the study.*

*Abstract*

*Define RDS on its first appearance*

**As suggested, we have added (RDS) after the instance where we mention respondent-driven sampling in the abstract, under Materials and Methods on line 34.**

*Main article*

*Page 7, line 122 dried blood spot (D\BS), I am not sure you wanted the back slash*

**We appreciate the reviewer catching this typo. We have deleted the “\” where indicated.**

*Page 7, line 125, Better to say viral load suppression but not undetectable. The lower limit detection of viral load for most machines is set at 50, 40, 20. At 1000, you still detect viruses*

**We thank the reviewer for this comment. We have eliminated mention of undetectable viral load, and changed the sentence in question to read: “Viral suppression was defined as <1000 copies/mL.”**

*Table1:*

*I will be better to rea arrange the columns such that*

*for 2013 arrange as 18-24 vs. 25 or older followed by its corresponding P-value. Do the same for 2016*

**For consistency with the other Tables and Figures in this manuscript, as well as with the text itself, we have opted to retain the use of ≥25 years, rather than changing Table 1 to read ’25 and older’.**

*Page 15 line 192: A couple of areas need to change verbs to past tense eg we compare, we see etc. This is true in some other areas as well*

**We have changed the tense of verbs in the present to past tense, per reviewer comment.**

*Page 15: There is title for the figure, however the figure is at the end. You may cite in the text and let the figure at the end, however, you also have tables in the text. Perhaps you should also move the figure up.*

**The placement of figures and tables are per Author Guidelines for this journal, therefore we have not made any change to address this comment. We are happy to submit in a different format if recommended by the editor.**

*Figure: Need to improve its quality*

**We have re-attached our figure with this re-submission. We believe the quality of our figure is per the standards of this journal, but please let us know if quality is an issue.**
